# Long-term stability of physiological signals within fluctuations of brain state under urethane anesthesia

**DOI:** 10.1371/journal.pone.0258939

**Published:** 2021-10-25

**Authors:** Nicholas R. G. Silver, Rachel Ward-Flanagan, Clayton T. Dickson

**Affiliations:** 1 Neuroscience and Mental Health Institute, University of Alberta, Edmonton, Canada; 2 Department of Psychology, University of Alberta, Edmonton, Canada; 3 Department of Physiology, University of Alberta, Edmonton, Canada; 4 Department of Anaesthesiology & Pain Medicine, University of Alberta, Edmonton, Canada; Belgrade University Faculty of Medicine, SERBIA

## Abstract

Urethane, an acute laboratory anesthetic, produces distinct neurophysiological and physiological effects creating an effective model of the dynamics of natural sleep. As a model of both sleep-like neurophysiological activity and the downstream peripheral function urethane is used to model a variety of physiological and pathophysiological processes. As urethane is typically administered as a single-bolus dose, it is unclear the stability of peripheral physiological functions both within and between brain-states under urethane anesthesia. In this present study, we recorded respiration rate and heart rate concurrently with local field potentials from the neocortex and hippocampus to determine the stability of peripheral physiological functions within and between brain-states under urethane anesthesia. Our data shows electroencephalographic characteristics and breathing rate are remarkable stable over long-term recordings within minor reductions in heart rate on the same time scale. Our findings indicate that the use of urethane to model peripheral physiological functions associated with changing brain states are stable during long duration experiments.

## Introduction

Urethane, an acute laboratory anesthetic, produces neurophysiological and physiological effects distinct from other anesthetics which make it an effective model of the dynamics of natural sleep. It is unique from other anesthetics in that it allows for spontaneous and cyclical alternations between a REM-like and an NREM-like brain state accompanied by corresponding changes in peripheral physiological signals that are also observed during natural sleep [1, 2]. While some anesthetics do produce NREM-like activity, no other anesthetic studied to date features the spontaneous cyclic changes between both brain and physiological states typically observed in natural sleep, at a surgical plane of anesthesia [1–5]. Due to these uncommon effects, urethane currently represents the best model of sleep, other than sleep itself. Accordingly, urethane has been adopted as a model of both sleep-like neurophysiological activity and the downstream physiological functions associated with changes in brain state, such as: activity-dependent neuroplasticity during slow-wave states; sudden, unexpected death in epilepsy; urodynamic functions associated with brain states; respiratory-related oscillations; brain and body temperature during sleep; pupillary associated changes with brain state; the role of astrocytes in brain state alternations; and sleep-disordered breathing [2, 6–24]

Urethane is further anomalous, in that it does not produce the severe cardiovascular or respiratory depression which are archetypal of most other anesthetics at a surgical plane [25]. The absence of this depression likely allows the physiological alternations accompanying brain state that are also observed in natural sleep. For example, heart rate is elevated during REM sleep as compared to NREM sleep and is likewise elevated during the activated (REM-like) state of urethane as compared to the deactivated (NREM-like) state [1, 26–28]. Furthermore, both breathing rate and breathing variability increases in REM sleep and the REM-like state in urethane [1, 23, 24, 29, 30].

Of importance is that a single bolus dose of urethane can produce a stable surgical plane of anesthesia for 6–24 hrs, with most research reporting at least 8 hrs of effective anesthesia [25, 31–33]. This level of anesthetic maintenance is advantageous, especially when compared to the negative impacts of infusing large quantities of other anesthetics over an extended period, and the added technical complications associated with continuous infusions [25, 32, 33]. However, despite the clearly established long-lasting nature of urethane anesthesia, it is unknown how stable individual physiological measures (within a particular state) remain across extended experimental recordings. Given that specific peripheral physiological measures, such as cardiac and respiratory rates, are important indicators of animal well-being during long duration procedures [25, 32], this is also an important ethical concern. Consequently, since urethane is an important model for a variety of sleep-like processes, it is imperative to fully characterize the longevity and stability of a variety of physiological measures to fully understand any potential limitations.

Here we document the long-term stability of a variety of physiological signals within and across brain state fluctuations during multiple hours of urethane anesthesia while animals were maintained at a surgical plane.

## Animals, materials and methods

Data was obtained from 6 male Sprague-Dawley rats weighing between 254 and 516g, averaging 360 ± 43g. Animals were initially kept on a 12 hour light/dark cycle at 20 ± 1⁰C, housed in cages of no more than 4 rats per cage. Cages were polycarbonate shoe-boxed shaped with wire tops, aspen wood chip bedding, and a PVC tube for enrichment. Standard rat chow and water were provided *ad libitum*. Welfare checks were preformed daily during housing before experiments. All methods were approved by the Biological Sciences Animal Care and Use Committee of the University of Alberta, conforming to the guidelines established by the Canadian Council on Animal Care.

### Anesthesia and surgery

Rats were initially induced in an enclosed chamber with gaseous isoflurane at a concentration of 4.0% mixed in 100% oxygen. Following a loss of righting reflexes, rats were maintained on isoflurane (2.0 to 2.5%) via a nose cone and implanted with a jugular catheter on the right side. Isoflurane was then discontinued and general anesthesia was achieved by slow intravenous administration of urethane (0.67g/ml; final dose 1.35g/kg). Body temperature was maintained at 37⁰C using a homeothermic monitoring system connected to a heating pad and rectal probe (Homeothermic Monitoring System, Harvard Apparatus, Holliston, MA) for the remainder of the surgical and recording procedures. Anesthetic plane was assessed throughout the experiment by monitoring for a reflexive withdrawal to a hind paw pinch.

### Stereotaxic procedures

Stereotaxic placement of bipolar recording electrodes was conducted using bregma as the landmark for coordinates. Recording electrodes were constructed from twisting a pair of Teflon-coated stainless steel wires (bare diameter 125 um: A-M Systems Inc., Sequim, WA). The two tips of these wires were staggered in length by 0.3–0.8mm. Two of these electrodes were placed in each rat, the first was in the neocortex (AP: +2.8; ML: +2.0; DV: -1.0 to -1.3 mm). The second target was straddling the CA1 pyramidal cell layer of the dorsal hippocampus (AP: -3.5, ML: -2.5, DV: -3.0 to -3.5 mm). Following implantation, the electrodes were subsequently fixed in place using a jeweler’s screw and dental acrylic. A thermocouple wire (30 gauge Type K; Thermo Electric Co., Inc.; Brampton, ON, Canada) was placed in front of the right nasal passage and shielded with aluminium foil. A pulse transducer (AD Instruments, Colorado Springs, CO) was attached to the right hind paw.

### Recording procedures

During the recording, the stereotaxic apparatus was connected to ground. Local field potentials and thermocouple signals were differentially amplified at a gain of 1000 and filtered between 0.1 and 500 Hz using an AC amplifier (Model 1700, A-M Systems Inc.). Amplified signals were recorded using a PowerLab AD board in conjunction with LabChart Pro (AD Instruments) and were sampled at 1000 Hz after anti-alias filtering. The thermocouple signal allowed for continuous online recording of breathing rate through measuring the difference in the temperature of inhaled and exhaled air. Heart rate was monitored throughout the recording period via the pulse transducer. Recording sessions lasted between 110–250 minutes. Following termination of the recording session, rats were transcardially perfused while under deep anesthesia.

### Data processing and analysis

Signals were first examined visually using LabChart Pro (AD Instruments) to segment data into specific recording periods. Files were further analysed using custom scripts for Matlab Version R2020a (Mathworks; Natick, MA) and processed using Origin Pro (Microcal Software Inc.; Northampton, MA).

Spectral analysis was accomplished using a series of 6-second long, Hanning-windowed samples with a 2-second overlap using Welch’s periodogram method. For spectrograms, a sliding window approach was used to analyze the data segment, wherein 30-second windows were moved across the data segment in 6-second increments. State changes could most reliably be characterized by large fluctuations in the power at 1Hz. Period analysis of these alternations was conducted by determining the saddle point of the bimodal distribution characterizing these power fluctuations, and this power value was used as a threshold for determining deactivated (>threshold) versus activated (<threshold) states.

Heart rate and breathing rate were analyzed using spectral time windows 20-seconds in duration, with a frequency resolution of 0.05 Hz. Peak frequencies were extracted and plotted across time for spectrographic analysis. Heart and breathing rate were compared both across and within states for any temporal variations throughout the duration of the experiment.

Summary reports of data across experiments and conditions were reported as arithmetic means together with standard error of the mean (SEM). Statistical comparisons were performed using paired t-tests, with a significance level of 0.05. Period length, ratio of activated to deactivated time, average heart rate, and average breathing rate were plotted as a function of time. Linear regressions were calculated using Prism 8 (GraphPad Prism Software Inc, San Diego, CA). The arithmetic means and standard deviations of breathing rate were calculated for each state over 1 minute in duration, and a coefficient of variation (standard deviation divided by arithmetic mean) was calculated from these values.

## Results

### EEG state properties and alternations are stable over long duration urethane anesthesia

**Fig 1:**
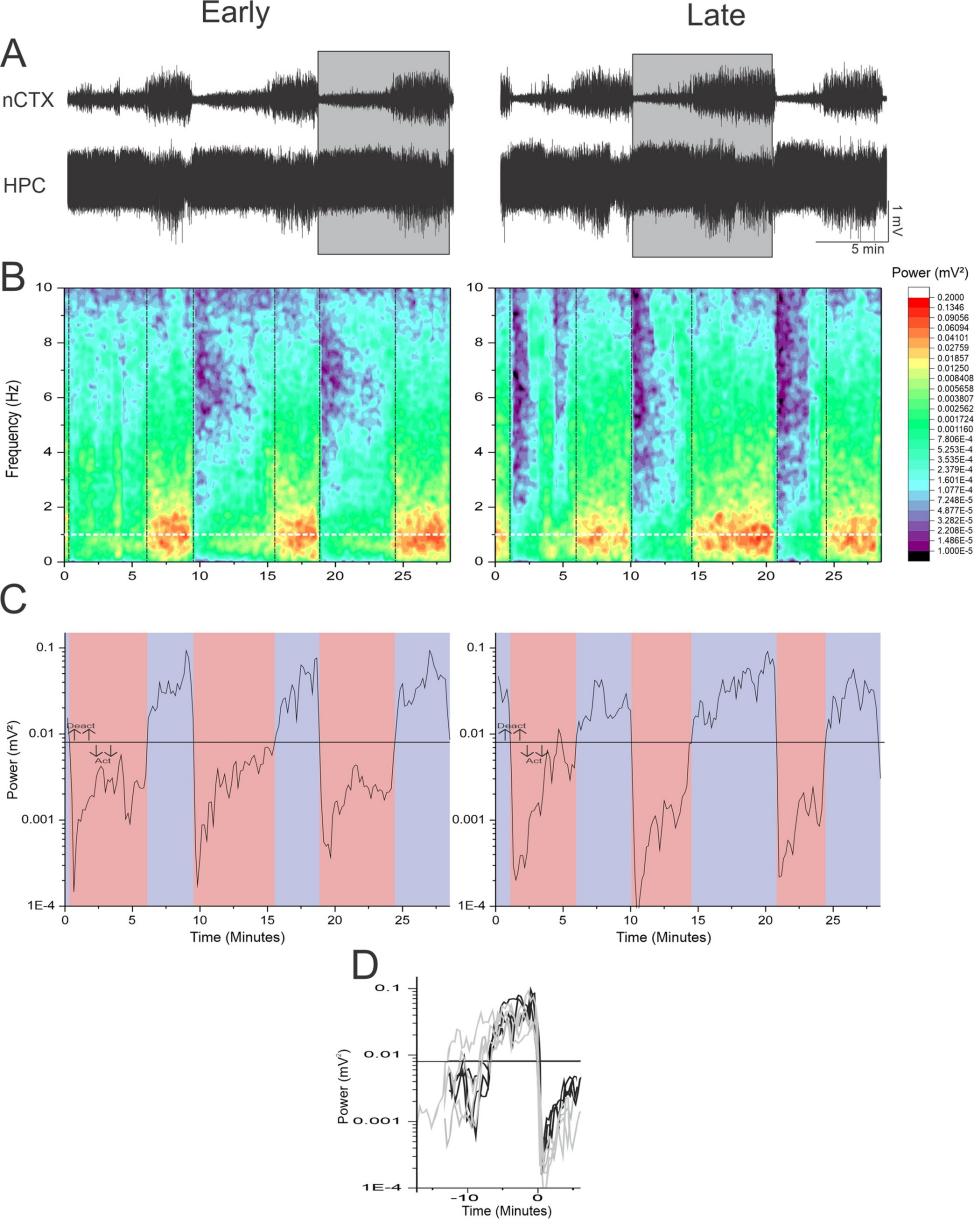
Spectral analysis of spontaneous cyclic alternations of cortical brain state under urethane anesthesia. **A)** 28.5 minute traces from the nCTX and HPC both early (starting 16 minutes) into the recording and late (starting 172 minutes) into the recording, depicting the spontaneous cyclic alternations seen in urethane anesthesia. Highlighted gray regions indicate the cycles used in Fig 2B. **B)** Spectrographic representation of the nCTX trace as shown in panel A. The most evident fluctuations were seen at ~1 Hz (indicated by the white dashed line). Alternation between states are indicated by the black dashed lines. **C)** Plot of 1 Hz power from the nCTX spectrograms (Panel B white dashed line) show cyclic fluctuations in amplitude corresponding to the transition between states. Deactivated states are denoted by the blue background, while activated states are denoted by the red background. **D)** Differing state change dynamics between the activated to deactivated and deactivated to activated state. Traces are taken form the 1 Hz power trace from the nCTX (Panel C). Selected period was from halfway through the proceeding activated state to halfway through the subsequent activated state. Time of 0 minutes was defined as time of transition form the deactivated to activated state. Transitions from the activated to deactivated sate were characterized by a more gradual variable transition, whereas transitions from the deactivated to active state were characterized by a rapid transition. Traces from the early in the recording are noted in black, whereas traces from the end of the recording are noted in grey.

**Fig 2:**
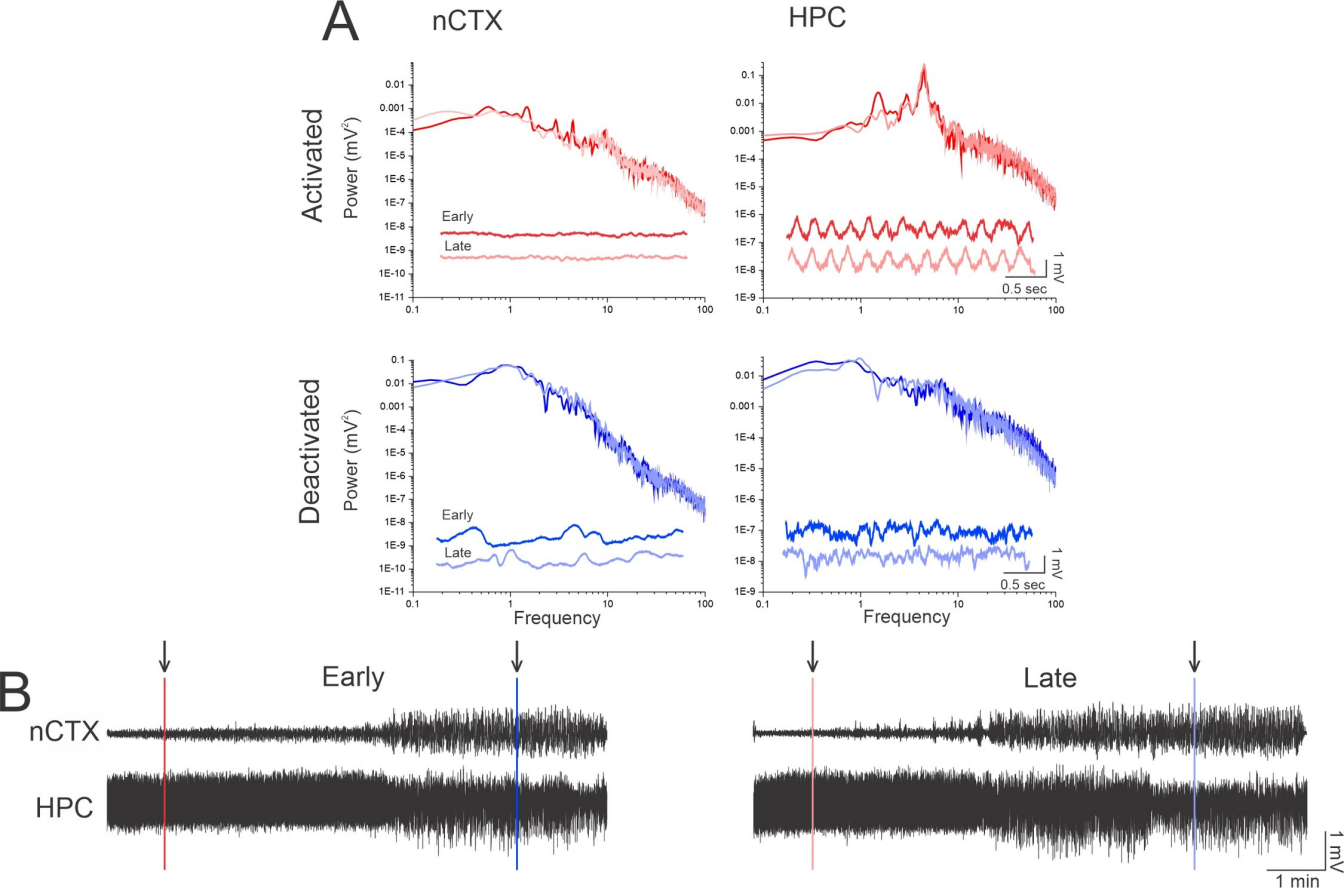
Spontaneous cyclic alternations of brain state under urethane anesthesia. **A)** Power analysis of the nCTX shows low voltage, higher frequency activity in the activated state both early (36 minutes) into the recording (dark red) and late (184 minutes) into the recording (light red). The HPC shows a strong peak corresponding to theta activity associated in the activated state both early (dark red) and late (light red) in the recording. Power analysis of the nCTX in deactivated shows a high voltage low frequency activity, associated with slow oscillations both early (43 minutes) into the recording and (dark blue) and late (190 minutes) into the recording (light blue) in the recording. The HPC shows high voltage, low frequency activity in the deactivated both early (dark blue) and late (light blue) in the recording. No major changes in power analysis is noted across states in both the nCTX and HPC in comparison of early and late recordings. Three second traces from the nCTX and HPC in both activated (red) and deactivated (blue) states are inserted in the corresponding power analysis. Early activated (36 minutes) in dark red and deactivated (43 minutes) in dark blue are the top traces. Late activated (184 minutes) in light red and deactivated (190 minutes) in light blue are the bottom traces. **B)** 9.5 minute trace of the nCTX (top) and HPC (bottom) early highlighting one cycle both (starting at 35 minutes into the recording) and a 10.5 minute trace of the nCTX (top) and HPC (bottom) late highlighting one cycle (starting 181 minutes into the recording). Lines and arrows indicate the location of the three second excerpts and where power analysis was taken for each state.

As previously demonstrated by our research group, (1) long-term local field potential recordings (EEG) taken from the neocortex (nCTX) and hippocampus (HPC) of urethane-anesthetized rats exhibited spontaneous, cyclic alternations in brain state between a deactivated (NREM-like) and activated (REM-like) state (Fig 1A–1C). The deactivated state was characterized primarily by large amplitude, low frequency (~1Hz) slow oscillatory activity in the nCTX and corresponding low frequency activity in the HPC (Fig 2A). Conversely, the activated state was distinguished by rhythmic higher frequency theta (~4 Hz) oscillations in the HPC and corresponding low amplitude fast activity in the nCTX (Fig 2A).

**Fig 3:**
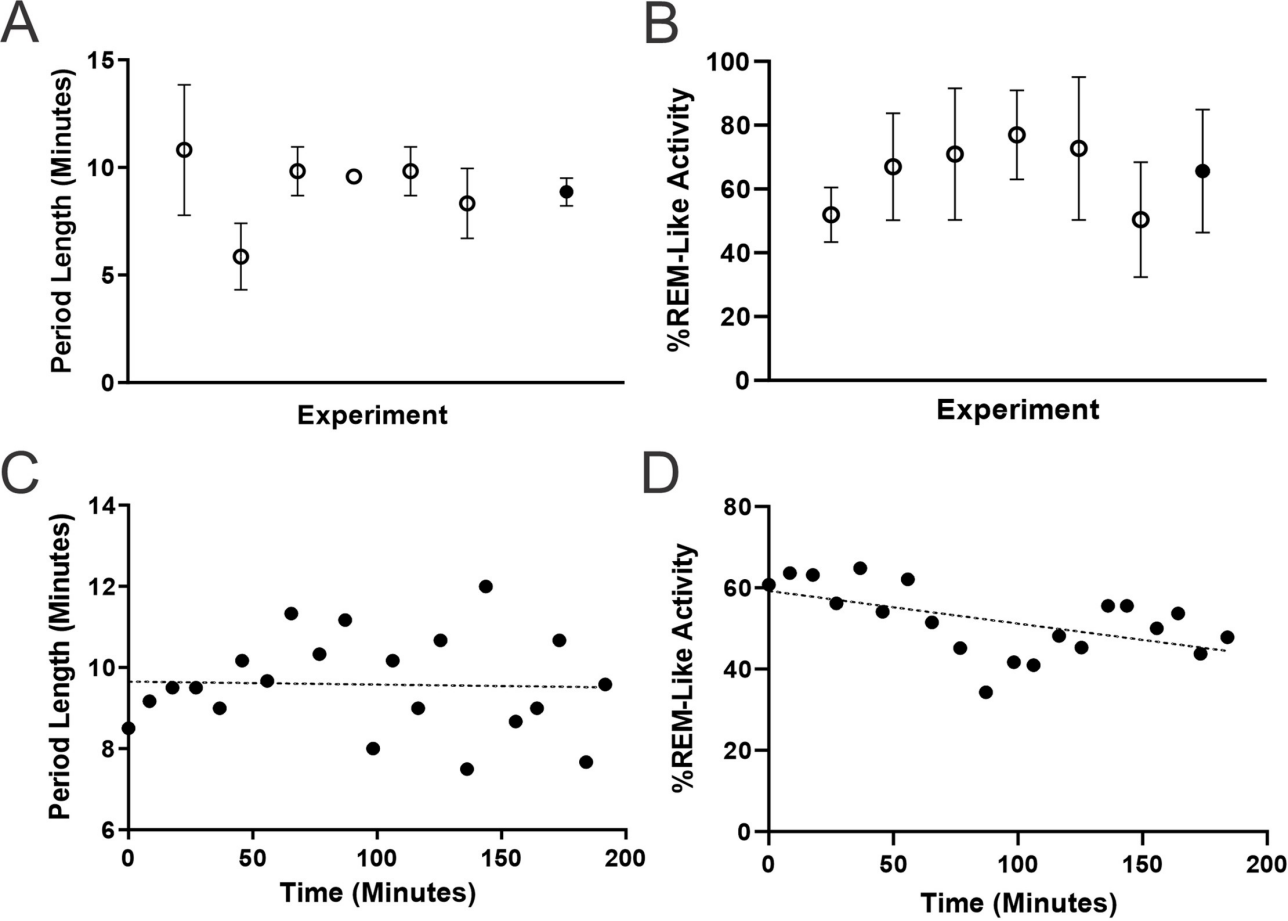
Stability of the spontaneous cyclic alternations in urethane anesthesia. **A)** The average period length (hollow circles) of individual experiments ranging in time from 110–250 minutes. Overall average period length (8.87±0.64 minutes) (solid circle) for all experiments. **B)** Percentage of REM-like activity relative to total period length for individual experiments (hollow circle) and overall experiments (65.6±0.02% REM-Like activity) (solid circle). **C)** Scatter plot showing period length as a function of time for a single experiment lasting 200 minutes. Linear regression line is shown in red and is not significantly different from zero (p = 0.8757), indicating the period length did not change throughout the experiment. Only 1 experiment had slopes significantly different from zero. **D)** Scatter plot of the percentage of REM-like activity relative to total period length for a single experiment. In this experiment, slope was significantly different than zero (R squared = 0.2953, p = 0.0133), however, this is the only experiment in which the percentage varied significantly with time.

State alternations were observed in all 6 animals tested, with an average period length of 8.87 ± 0.64 minutes (Fig 3A). Within this period, the average length of the activated state was 5.91 ± 0.49 minutes, while the average length of the deactivated state was 2.97 ± 0.29 minutes. The cycling between these distinct states appeared to be stable throughout long-term recordings, as seen in both the raw EEG traces and spectrograms of data segments throughout extended (~3-hour) recording sessions (Fig 1A–1C). Fluctuations were most prominently characterized by variations in spectral power at 1 Hz. Extracting this frequency band and plotting it across time highlighted the periodicity and rhythmicity of the state alternations observed under urethane (Fig 1C). As shown, this periodicity was highly consistent across this extended timeframe. In 5 of the 6 animals, the period length did not differ throughout the entire recording time. In the one remaining experiment, the period length showed a slow but significant decrease with time as calculated by regression (R-squared = 0.405, p = 0.0112), with an average slope of -5.86 ± 1.99 min/hr. Another feature of the transition between the activated to deactivated state was that the power at 1 Hz tended to show a gradual build up over time, whereas the transition from deactivated to activated was a more abrupt change over a shorter period of time (Fig 1A–1D). These dynamics were also maintained throughout the recording session (Fig 1D).

The percentage of time spent in the activated state in relation to overall period length was used to determine if the period composition changed as a function of time. The average period was 65.6 ± 0.02% REM-like activity (Fig 3B). The amount of REM-like activity only varied with time in one experiment, with a slope of -8.0 x10^-4^ ± 3.0 x10^-4^ (R squared = 0.2953, p = 0.0133) indicating an increasing amount of time spent in the NREM-like state as the recording progressed (Fig 3D).

### Breathing rate measures within states were consistent over long duration urethane anesthesia

**Fig 4:**
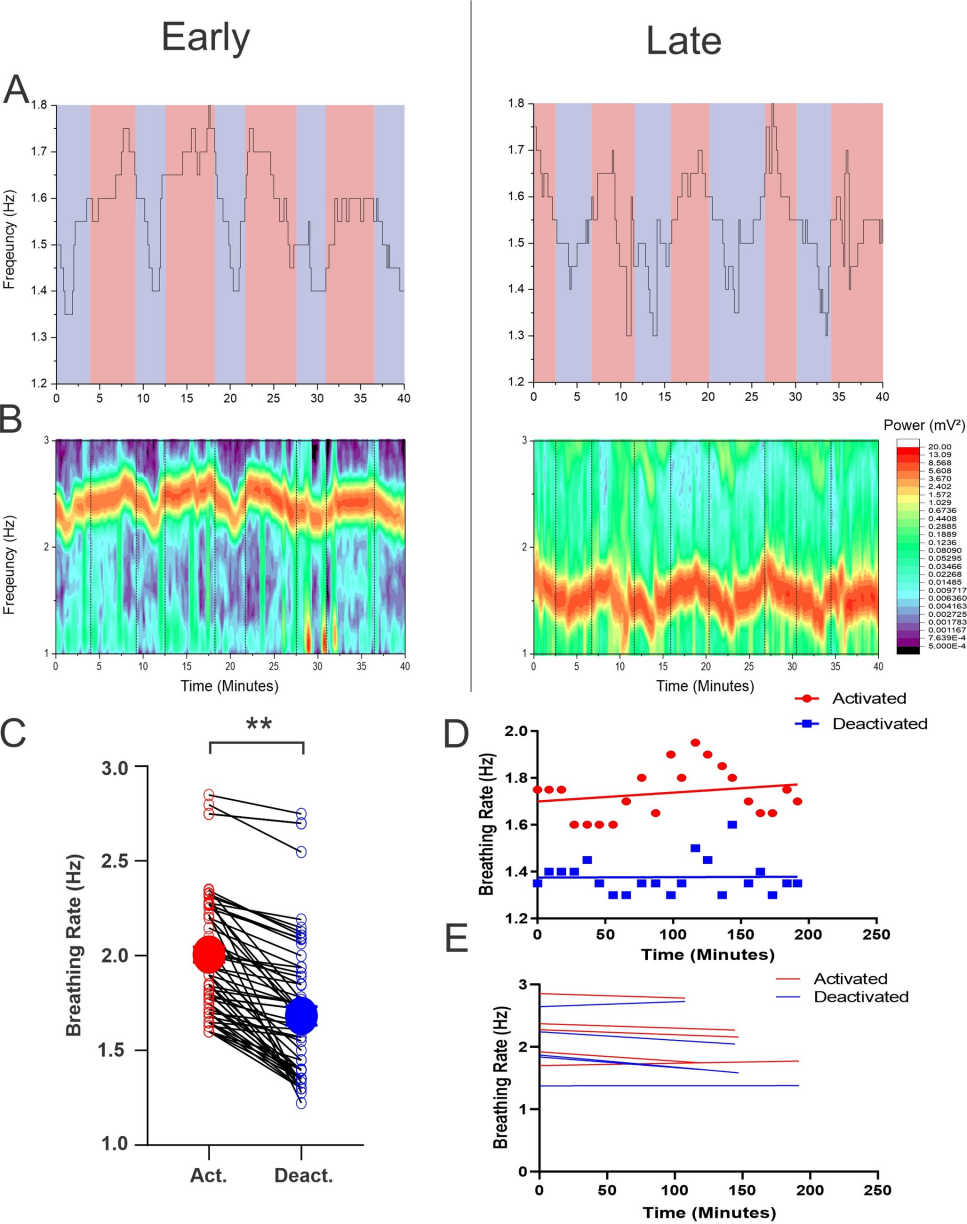
Stability of breathing rate under urethane anesthesia. **A)** Extraction of peak frequency of peak breathing rate during the first 40 minutes (left) and last 40 minute (right) of a 200-minute recording session. Deactivated states are indicated by the blue background, and activated states are indicated by the red backgrounds. Breathing rate remained stable and did not decrease as heart rate did. Fluctuations corresponding to activated and deactivated states remain stable throughout the recording session. Note the increase variance during the activated state. **B)** Spectrographic analysis of breathing rate showing the first 40 minutes (left) and last 40 minutes (right) of a 200 minute recording session. Fluctuations between activated and deactivated are indicated by black dashed lines. Fluctuations were primarily seen at ~2.0 Hz (120 breaths per minute). **C)** Overall average breathing rate for the activated (red circle) (2.00 ± 0.040 Hz) and deactivated (blue circle) (1.67 ± 0.048 Hz) states for all experiments. A 0.32 Hz (95% CI: 0.27 to 0.37 Hz) decrease was seen between the activated and deactivated state, this difference is significant (t(59) = 14.06, p < 0.0001). Dot and lines show all transitions. **D)** Activated (red) and deactivated (blue) breathing rates plotted against time for a single experiment with their respective linear regressions. Neither deactivated nor activated breathing rate different significantly from zero (p = 0.9540, p = 0.3478). Note the greater variation of breathing rate within the activated state (CoV, activated = 0.045±0.0054, CoV, deactivated = 0.024±0.0030, t(59) = 4.56, p<0.0001). **E)** The breathing rate of the activated (red) and deactivated (blue) states as a function of time for all experiments.

Breathing rate was monitored in 5 out 6 animals, as signal dropout was an issue in one rat. As our research group has previously shown, respiration frequency exhibited variability corresponding to the brain state of the animal [1]. The activated state coincided with both increased rate and inter-period variability, while the deactivated state corresponded to a lower frequency, but more uniform breathing rate. This is seen in both spectrogram and peak frequency analysis of breathing rate (Fig 4A, 4B). The deactivated state had an average breathing rate of 1.67 ± 0.048 Hz with a coefficient of variation (CoV) of 0.024 ± 0.0030, while the activated state gave an average breathing rate of 2.00 ± 0.040 Hz with a higher CoV of 0.045 ± 0.0054. The difference in breathing rate between the states corresponded to ~20 breaths per minute (0.32 Hz; 95% CI: 0.27 to 0.37 Hz). This was a statistically significant change (t(59) = 14.06, p < 0.0001) (Fig 4C). As well, the difference between the CoV measures across states was also significant (t(59) = 4.56, p<0.0001). These fluctuations were preserved throughout a long-term recording session. In all experiments breathing rate within a unique state did not change as a function of time (Fig 4E).

### Heart rate measures within states were consistent over long duration urethane anesthesia

**Fig 5:**
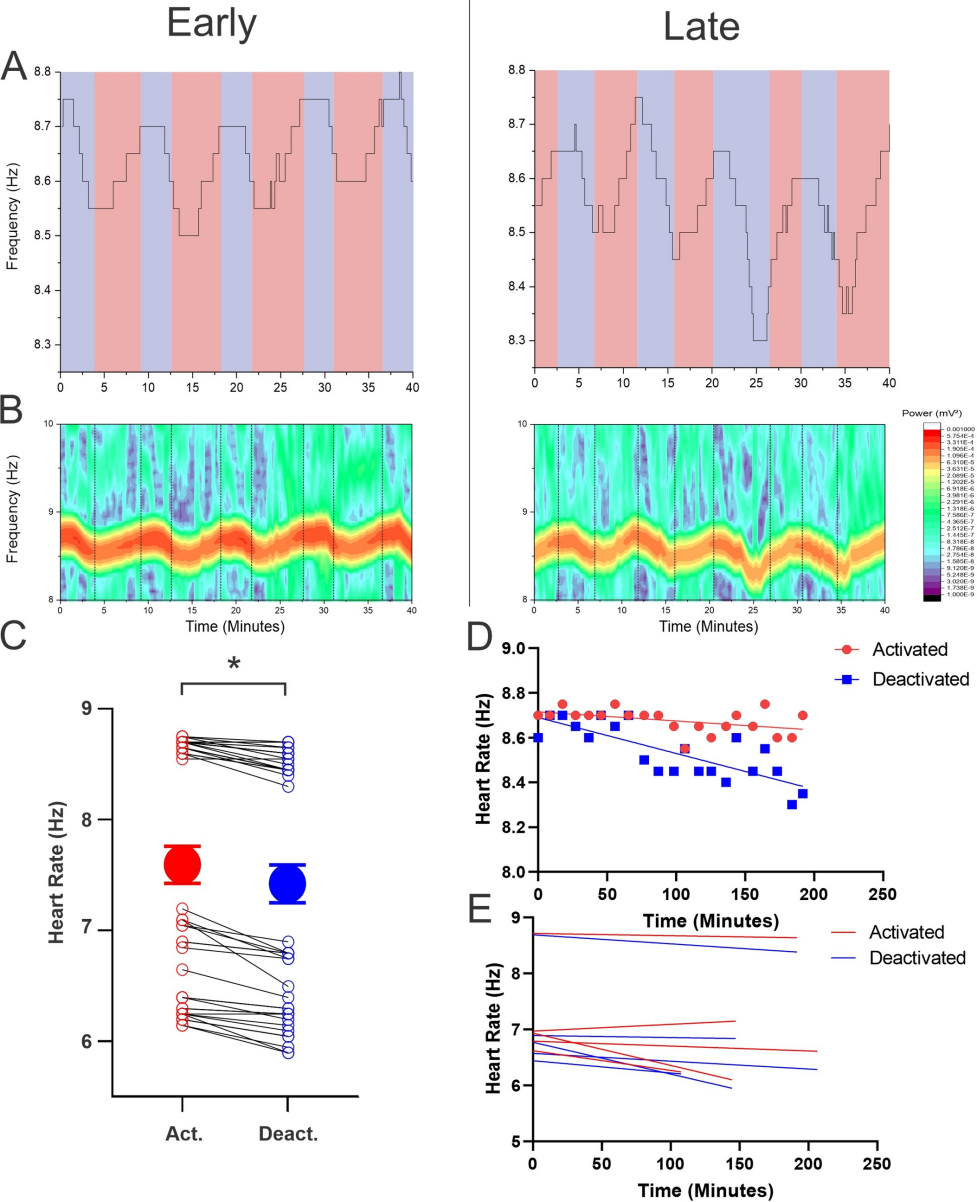
Stability of heart rate under urethane anesthesia. **A)** Extraction of peak frequency of peak heart rate during the first 40 minutes (left) and last 40 minutes (right) of a 200 minute recording session. Deactivated states are indicated by the blue background, and activated states are indicated by the red backgrounds. While fluctuations corresponding to activated and deactivated remain, the heart rate decreased throughout the recording period. **B)** Spectrographic analysis of heart rate showing the first 40 minutes (left) and last 40 minutes (right) of a 200 minute recording session. Fluctuations between activated and deactivated are indicated by black dashed lines. Fluctuations were primarily seen at ~8.6 Hz (516 beats per minute). **C)** Overall average heart rate for the activated (large red circle) (7.60±0.17 Hz) and deactivated (large blue circle) (7.42±0.17 Hz) states for all experiments. A 0.17 Hz (95% CI: 0.1381 to 0.206100) decrease was seen between activated and deactivated, this difference was significant (t(59) = 9.04, p < 0.0001). Dot and lines show all transitions. **D)** Activated (red) and deactivated (blue) heart rates plotted against time for a single experiment with their corresponding linear regressions. Activated heart rate was significantly different then zero (R squared = 0.2070, p = 0.0383) as well as the deactivated heart rate being significantly different from zero (R squared = 0.6276, p<0.0001). **E)** The heart rate of the activated (red) and deactivated (blue) states as a function of time for all experiments.

Heart rate was monitored in 5 animals, as heart rate could not be recorded due to technical issues in one animal. As we have previously reported, heart rate also showed fluctuations that corresponded to the brain state of the animal [1]. The activated state coincided with an increased heart rate, while the deactivated state corresponded to a decreased heart rate as shown in both peak frequency and spectrographic analysis (Fig 5A, 5B). These related electrophysiological fluctuations were conserved throughout a long-term recording session. The deactivated state had an average heart rate of 7.42 ± 0.17 Hz, while the activated state had an average heart rate of 7.60 ± 0.17 Hz. The difference between the two states was 0.17 Hz (95% CI: 0.1381 to 0.206100) or ~10 beats per minute; this difference was statistically significant (t(59) = 9.04, p < 0.0001) (Fig 4C).

We also assessed the degree of stability of heart rate measures within states as a function of time spent anesthetized. As a function of time, heart rate decreased within both states (Fig 5D). The heart rate in the activated state decreased with time in 4 out of 5 experiments, with an average slope of -2.6x10^-3^ ± 1.3x10^-3^ Hz/minute, the slope of these experiments was not significantly different. The same decreasing pattern was observed in the deactivated state over time in the same four experiments in which the activated heart rate decreased. The heart rate of the deactivated state as a function of time on average had a slope of -2.7 x10^-3^ ± 1.0 x10^-4^ Hz/minute or roughly 0.16 beats/minute. The slope of these experiments were not significantly different from each other.

## Discussion

Our results reveal the long-term stability of critical physiological functions associated with the sleep-like brain state alternations that are characteristic of urethane anesthesia. We observed that sleep-like forebrain state alternations under urethane remained stable in both periodicity and alternation dynamics across multiple (in most cases, ~3) hours of a single dose of urethane anesthesia. Monitoring of respiration throughout the recordings demonstrated consistent changes in rate and variability concomitant with changes in brain state, resembling transitions seen in natural sleep [30, 34]. A minor depression of respiratory activity over the entirety of recordings was observed, but was equivalent with the respiratory depression typically seen in natural sleep [1, 29, 30]. State-dependent differences in heart rate remained stable throughout the recording sessions and were also comparable to those observed in natural sleep [1, 35]. The stability of all evaluated physiological signals over extended recording sessions indicates that urethane can produce sustained neurophysiological and physiological alternations characteristic of a sleep-like state over multiple hours.

### Stability of peripheral physiological measures

Spontaneous, cyclic alternations in forebrain state with accompanying peripheral state-dependent modulation of respiration and heart rate were stable across the extended time frame of the recordings. The stability of these characteristics, at a surgical plane of anesthesia, indicates that the extent of the metabolism of urethane that occurred during the duration of the experiments was not sufficient to alter the ongoing neurophysiological dynamics, nor accompanying physiological changes. This demonstrates that a remarkably consistent sleep-like state is produced by urethane. While these stereotyped physiological signals were only recorded for ~3 hrs in this study, previous studies indicate the effective duration of anesthesia from a single dose of urethane to be on the order of at least 8 hrs [25, 31–33]. Therefore, it is probable that these stereotyped physiological characteristics are present and stable even beyond the duration of what we have reported here.

While respiration measures within states were stable throughout the entire duration of our recordings, heart rate did show a modest decrease as a function of time, which could be explained by the negative inotropic effects and subsequent suppression of cardiac rhythms, due to the concentration of urethane necessary for maintaining a surgical plane of anesthesia [36]. Despite this modest cardiovascular effect over time, it has been previously demonstrated that urethane still allows for the maintenance of cardiovascular reflexes, such as vagal nerve reflexes, making it a suitable candidate for the assessment of cardiovascular responses [36].

### Central neurophysiological and peripheral physiological similarities between urethane and natural sleep

Our data shows that the forebrain state dynamics under urethane are maintained for long durations, verifying that urethane is a suitable model of sleep over long recording periods. On average, the activated and deactivated brain states in rats under urethane anesthesia cycle with a consistent and stereotyped period of 10.1–13.2 minutes [1, 30, 37]. This period replicates the dynamics observed during natural sleep in rats, which has a distribution of 9–13 minutes [34]. Our current study demonstrates that the time spent in each brain state and the associated cycling between states is consistent throughout long-term recordings (Fig 3). While this overall consistency is dissimilar to natural sleep, which has progressively less NREM activity per period as sleep progresses, this is arguably beneficial for experimental paradigms that require stability and predictability over extended periods of time [38, 39]. Such consistency allows researchers to determine the effects of experimental manipulations on both time spent in each sleep-like brain state, and consistency of alternations between states [29, 37, 40–42].

Respiratory rate and heart rate under urethane anesthesia exhibit similar dynamics to that of natural sleep. NREM sleep is characterized physiologically by a depressed breathing rate of ~1.6 Hz, and a correspondingly depressed heart rate of ~5.5 Hz [26–28, 35, 43–46]. During the deactivated state under urethane anesthesia, we observed an average breathing rate of 1.67 Hz, analogous to the respiratory depression in NREM, and a heart rate of 7.42 Hz. It is important to note that heart rate during sleep is extremely variable depending on the age, species, and circadian rhythm of the animal and as such, the heart rate observed under urethane anesthesia is not outside the range of reported values in natural sleep [44–46].

REM sleep is characterized by an elevated breathing rate of 1.8–2.0 Hz and a greater degree of respiratory variability, together with an elevated heart rate of ~5.6 Hz [26–28, 35, 43–46]. During the activated state under urethane anesthesia, the respiratory rate averaged 2.0 Hz, and also showed greater periodic variability compared to the deactivated state. Heart rate was also found to be elevated in this state relative to the deactivated state with an average heart rate of 7.6 Hz in the activated state. These sleep-like changes in respiratory rate, respiratory variability, and heart rate are maintained throughout long-term recordings under urethane anesthesia as would be expected in natural sleep.

### Summary

Urethane allows for dynamically changing central and peripheral physiological signals which parallel natural sleep. We have found that within each brain state, these neurophysiological signals are highly stable across long periods of time. Moreover, across long-term urethane anesthesia the dynamics of these changing physiological signals are highly stereotyped and allow for tractable neurobiological and drug manipulations. Therefore, the long-term stability of urethane represents a powerful tool for exploring sleep-like changes in physiological function, whether central or peripheral, in acute experimental paradigms where natural sleep is either technically challenging or ethically intractable.
